# Risk multipliers for severe food anaphylaxis

**DOI:** 10.1186/s40413-015-0081-0

**Published:** 2015-11-24

**Authors:** Peter K Smith, Jonathan O’B Hourihane, Phil Lieberman

**Affiliations:** Griffith School of Medicine, Gold Coast, Australia; University College Cork, Cork, Ireland; University of Tennessee College of Medicine, Memphis, Tennessee USA

## Abstract

Anaphylaxis is a severe, life threatening allergic reaction. In most fatal cases of food anaphylaxis, the fatality is not due merely to a simple, linear relationship between the allergen and exposure in a sensitized individual. Compounding factors such as the allergic disease burden—particularly the presence of asthma; comprehension of the potential severity of an event, training in the appropriate use of epinephrine, and emerging metabolic factors should be considered when assessing risk and establishing management strategies. This paper reviews the factors that contribute to the risk of severe anaphylactic events and provides a framework for the ongoing management of patients at risk of severe food allergy.

## Review

Death from food anaphylaxis remains a rare but tragic event [1]. The majority of fatal food anaphylaxis involves children and young adults [1–3]. In most fatal cases there is a combination of several known risk factors, which often could have been individually mitigated. The purpose of this review is to highlight known factors that increase the risk for severe allergic reactions to food and to present, in a practical and visual manner, strategies that can be used to identify and deal with these factors when taking a history, examining and planning management for individuals with food allergy.

Anaphylaxis is a serious, life-threatening, generalized hypersensitivity reaction that can occur via immunologic (either IgE-dependent or IgE-independent) or non-immunologic mechanisms [4]. Effective management of potentially life-threating reactions involves review by an allergist to identify causative allergens (and other allergic risks to help reduce exposure), and the prescription of and training in the use of epinephrine [5]. This referral for an evaluation should best occur at the emergency care setting where, currently, under-diagnosis of anaphylaxis is an issue [6]. The following aspects need to be highlighted as particular risk factors for anaphylaxis, with an emphasis on food anaphylaxis.

### Dose and type of allergen

With IgE dependent reactions it is considered axiomatic that the dose of allergen that the sensitized individual is exposed to determines the severity of the reaction [7, 8]. However it should be appreciated that a threshold dose for symptoms can vary for an individual and between individuals. The concealment of allergen (hidden allergen) can result in delayed recognition of an allergic food and increase dose exposure. The median time from ingestion of peanut to experiencing symptoms has been reported to be as short as 3 minutes [9]. In adult subjects undergoing repeat peanut challenges, an increase in the lipid matrix of a concealed peanut biscuit led to higher dosing before the patient developed symptoms, and consequently they experienced more severe allergic and anaphylactic reactions [10]. Other concealment/dosing factors may include spicy foods where itch and burning from spices could mimic allergic symptoms. Case series have reported an apparent effect of alcohol/sedating medication consumption, where symptom recognition is reduced, though there are no published studies on dose-thresholds related to these latter factors. The type of allergen contributes to concealment and exposure risk. In a threshold dose study, peanut caused more severe reactions than other foods studied (hazelnut, egg and milk) [11]. In a study investigating the correlation of dose with severity of the reaction in a community setting and the threshold dose of peanut in a double blind placebo controlled challenge, the amount of the dose did not relate significantly to either the severity of the reaction or the dose of peanut reportedly consumed in the community [12]. This suggests that there are very important co-factors that influence the severity of food allergic reactions outside the controlled clinical setting of a formal food challenge [13].

### Age

Youth—specifically teenage years and being a young adult—is a risk factor for fatal reactions [1–3, 14, 15]. The expected attributable social and emotional contributing factors include risk-taking behavior with eating, disease denial or treatment non-compliance (not carrying an epinephrine auto-injector and poor asthma control) [16]. Although not specific to food allergy, Brown et al. reported that older age was associated with more severe hypoxemia with anaphylaxis episodes [17]. In a peanut threshold dose study, adults with peanut allergy had more severe reactions than children [11]. This finding has very significant implications, for the majority of patients with peanut allergy do not grow out of their sensitivity.

### Late or absent treatment

Failure to use epinephrine, or to use it promptly is considered to be an important and avoidable factor in fatal reactions [5, 17–21]. Combining the data of Bock and Pumphrey in cases of fatal food anaphylaxis, only 5 of 80 patients had administered epinephrine appropriately [2, 3]. Intramuscular epinephrine in the community setting is the standard of care for anaphylaxis, as inhaled epinephrine devices are unreliable in children [22] and subcutaneous epinephrine takes up to 30 minutes to achieve significant serum levels [23, 24].

### Asthma

Asthma is probably the most significant risk factor for death from food allergy anaphylaxis, with case series finding 69–100 % of patients having pre-existing asthma [2, 3, 5, 13, 18–21, 25]. Summers and colleagues reported that allergic burden contributes to the severity of allergic reactions in patients with nut anaphylaxis [26]. In patients with severe asthma the risk for life-threatening bronchospasm during nut associated anaphylaxis was increased 6.8 fold, although this relative risk was only 2.7 times for those patients with mild asthma. With active allergic airway disease, an increase in mast cells, basophils, neutrophils and eosinophils appear in the airways [27–30]. Cornell described a clinical entity of airway “priming” [31, 32], where pollen-allergic patients experience a greater allergic reaction (and with smaller doses of allergens) at the end of the pollen season than at the start. Priming has been demonstrated experimentally [27, 28] and can be reduced with the use of nasal steroids [33]. Priming increases symptoms to both the priming antigen as well as non-priming antigens and non-allergic stimuli [32]. We hypothesize that the Summers et al. report, reflects increased effector cell activity with allergic disease burden due to “priming”, which resulted in more severe food allergy reactions. Swedish children with inhalant allergies suffer food anaphylaxis more commonly in their relevant pollen season, compared to children without pollen allergy, whose event rate is more stable through the year [34]. Oral allergy syndrome (also called secondary food allergy or pollen-food allergy) may increase in severity in high pollen season [35]. Whilst oral allergy syndrome is often regarded as mild disease, severe reactions including anaphylaxis can occur [36].

### Past history of severe allergic reactions

A history of severe allergic events including anaphylaxis has been identified as a risk factor for fatal events due to food and future severe allergic reactions [19, 37]. A history of anaphylaxis also appears to influence both the dose of an allergen tolerated in patients receiving food allergy oral immunotherapy and the risk of severe reactions during oral food immunotherapy [38]. It should be stressed that the about half of a UK series of food anaphylaxis deaths occurred in patients with a history of mild reactions, thus there can be little reassurance based on a history of previous mild reactions [39].

### Allergic disease burden

In a case series of patients with nut allergy, severe rhinitis was associated with a 3.8 fold increased risk for severe pharyngeal edema and severe eczema correlated with a 3.1- fold increased risk of impaired consciousness (presumably hypotension) with anaphylaxis events [26]. Although aging is physiological rather than a “disease”, increasing age has been correlated with a higher risk of severe cardiovascular symptoms [17, 40].

### Physiological and medication

Multiple allergic mediators (e.g., tryptase, histamine, platelet activating factor, IL-2, IL-6, IL-10 and soluble tumor necrosis factor receptor I), have been correlated with severity of anaphylactic reactions [17, 39-41]. These mediators are products of the allergic reaction, rather than being risk factors for anaphylaxis.

Variants in the catabolic capacity of two enzymes have been correlated with risk for severity of anaphylaxis [17, 26]. Patients with serum angiotensin converting enzyme l (ACE) levels in the lower quartile of normal (<37.0 mmol/L) have been reported to have a 9.6 (1.6–57) - fold risk of severe pharyngeal edema when experiencing anaphylaxis from a nut source compared to those in the higher quartile [26]. In a study of all forms of anaphylaxis, platelet activating factor acetyl hydrolase activity (also known as Lipoprotein-associated phospholipase A2) was found to be associated with severe reactions in a proportion of patients [17]. Serum PAF levels have also been linked to anaphylaxis severity [42]. Platelet activating factor acetyl hydrolase is responsible for PAF degradation in plasma [43]. ACE is the main protease responsible for bradykinin catabolism [44]. There are two allelic forms of ACE, and increased expression of the gene for the “I” allele has been associated with more severe food allergy [45].

While medications cause most healthcare related episodes of anaphylaxis, only aspirin has been reported to be related to food dependent exercise induced anaphylaxis [46, 47]. Beta-blockers, cox-inhibitors and ACE inhibitors have been reported as possible contributors to the severity of all forms of anaphylaxis [13, 40], and both Beta-blockers and ACE inhibitors have been associated with mast cell priming [48]. Gliptins (which are used in diabetes and inhibit a serine protease, have been reported to augment ACE induced angioedema [49]. Aspirin may cause increased gastrointestinal permeability, and therefore the rapidity and amount of allergen levels reaching the submucosa/systemic circulation. A 500 mg dose of aspirin has been reported to increase the likelihood of a positive food challenge in patients with wheat dependent exercise induced anaphylaxis [47]. While not specific for food, Brown et al. reported that use of cardiovascular medications correlated with age but was not associated with additional risk for reaction severity [17].

Menstruation may be another physiological state that destabilizes the allergic status of an individual and the relation of anaphylactic episodes to the menses should be considered when appropriate.

### Exercise

Exercise can cause anaphylaxis directly and is a co-factor for food anaphylaxis, best defined as food dependent exercise induced anaphylaxis (FDEIA). Wheat-derived omega 5 gliadin is the antigen most commonly recognized as causing this syndrome [50, 51]. A US series reported shellfish crustaceans to be the allergen in 37 % of cases of FDIEA [52]. Because of the multi-factorial nature of this condition, the diagnosis can be difficult to make (the patient often experiences multiple severe attacks over a prolonged time) because the patient can tolerate the food remote from exercise without symptoms. It is proposed that exercise increases GI permeability and/or allergen digestion, so the patient is exposed to greater levels of antigen [35, 53]. Exercise is a physiological state that increases release of mediators (serotonin, bradykinin and endorphins), which sensitize the calcium ion channel through which histamine works, the transient receptor potential vanilloid 1 (TRPV1) receptor [54]. This ion channel is present on both sensory nerves and mast cells. Acidosis and rise in core body temperature also directly agonize the TPRV1 ion channel to further reduce the activation threshold to allergic products [54]. ATP, which increases with exercise, inhibits the homeostatic de-activation of TRPV1 receptor [55].

### Intercurrent illness

Infective illness has similar metabolic influences as exercise, and there is evidence of immunological vulnerability including mast cell activation [56]. The best data relating to food allergy and a reduced threshold for food with infective illness comes from food allergy oral immunotherapy reports, where doses of tolerated allergen have a higher rate of reaction during illness [57]. Hot showers have also been reported to be associated with risk of reaction to food allergens during immunotherapy [38]. Medications such as aspirin used to control symptoms and fever with infections might also contribute to risk of severe allergic reactions to food.

### Comprehension and education

Anecdotal reports suggest there may be an effect of ethnicity on the incidence of allergic reactions to foods though it is unclear how this is mediated; it is possibly due to language and cultural difficulties inhibiting equivalent access to allergy focused clinical services and optimization of asthma care.

Education provided regarding food allergy and anaphylaxis will enhance the prevention, recognition and appropriate and timely therapy of anaphylactic reactions to food. The authors are aware from coroners' reports that learning difficulty (e.g., intellectual disability and language barriers) have been found to be issues in food anaphylaxis deaths.

### Other factors

Many allergists recognize ethanol as risk factor for food allergy anaphylaxis, however, there is very little published literature on this [58, 59]. Ethanol, like other recreational drugs, can be associated with reduced awareness, risk taking, and recognition of symptoms, as well as interfering with an individual’s timely treatment response to severe food allergy. Physiologically, ethanol activates the TRPV1 channel, lowering the threshold to activation by the multiple endogenous products of the allergic response that act via this ion channel [54, 60]. Activation of the TRPV1 ion channel on sensory nerves results in release of neuromediators including calcitonin nerve related peptide to cause vasodilation [61]. The vasodilation effect from ethanol could contribute to a more severe shock in food allergy. Alcohol may also cause gastritis, permitting greater allergen absorption.

Mastocytosis/mast cell tumors/mast cell activation should be included in underlying risk factors for severity of anaphylaxis in general, however, we are not aware of a food allergy death being reported in a patient with documented mastocytosis/mast cell activation syndromes.

## In summary

**Fig. 1:**
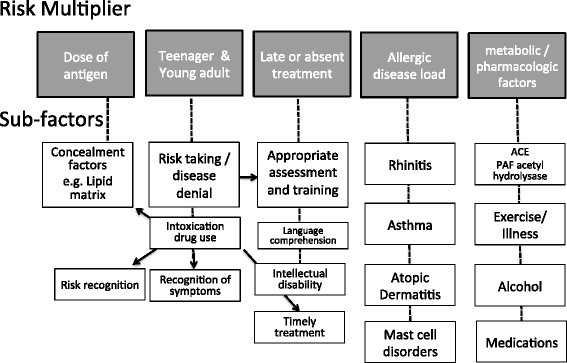
Layers and multiplication factors relating to risk in severe food allergy: 1. Dose of antigen, which can be altered by concealment factors, 2. Age—most deaths from food allergy are in the young, but older patients tend to have more severe reactions with nut allergy, 3. Timely effective treatment, 4. Co-existent allergic burden, and 5. Metabolic/Pharmacologic factors including: capacity to catabolize allergic mediators, exercise, illness, ethanol and medications.

**Fig. 2:**
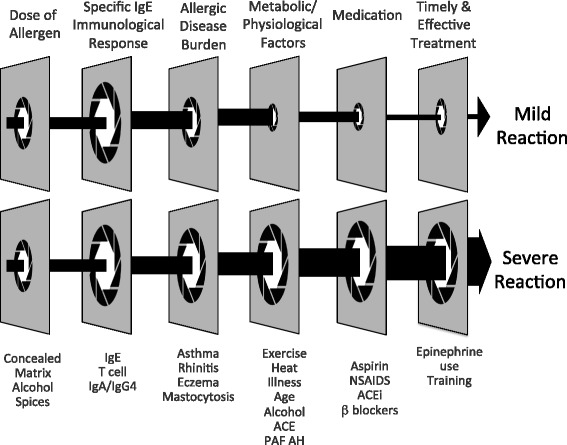
An allergic individual's response to an allergen may vary because of dose, treatment and threshold factors. Timely, effective treatment limits, but does not control, all reactions. Apertures are larger depending on the presence of risk factors. Fatal and severe reactions appear more likely if there is a combination and alignment of risk factors. In the example, a similar dose in patients with equivalent levels of severe food allergy has different clinical outcomes. A mild reaction is the outcome in a patient with less current allergic disease, less metabolic factors, less contributing medications and early effective use of epinephrine. These factors are shown to amplify in a severe allergic reaction

There are many layers to the risk of severe reactions in food allergy. A model of augmenting factors has recently been suggested [62]. We have developed 2 frameworks to consider risk factors. These co-contributors are summarized in Fig. 1. More severe and fatal reactions are likely to occur with alignment of these layers-the so-called “swiss-cheese” model, and a gap emerges. A larger aperture to “fall through” opens with more risk factors, and the result is likely to be a severe reaction if there is alignment (Fig. 2). The role of the allergy specialist is critical in risk identification, the communication of risk, treatment of co-morbidities and having a supportive, empowering and effective management plan in place with medications on hand for patients and families to self treat when accidentally exposed to food and other allergens.
